# COVID-19 Contact Tracing Outcomes in Washington State, August and October 2020

**DOI:** 10.3389/fpubh.2021.782296

**Published:** 2021-11-26

**Authors:** Robert A. Bonacci, Lillian M. Manahan, James S. Miller, Patrick K. Moonan, Missy B. Lipparelli, Lisa M. DiFedele, Lora B. Davis, R. Ryan Lash, John E. Oeltmann

**Affiliations:** ^1^Epidemic Intelligence Service, CDC, Atlanta, GA, United States; ^2^COVID-19 Response Team, CDC, Atlanta, GA, United States; ^3^Washington State Department of Health, Tumwater, WA, United States

**Keywords:** COVID-19, contact tracing, case investigation, public health, surveillance

## Abstract

**Introduction:** Case investigation and contact tracing are important tools to limit the spread of SARS-CoV-2, particularly when implemented efficiently. Our objective was to evaluate participation in and timeliness of COVID-19 contact tracing and whether these measures changed over time.

**Methods:** We retrospectively assessed COVID-19 case investigation and contact tracing surveillance data from the Washington State centralized program for August 1–31, 2020 and October 1–31, 2020. We combined SARS-CoV-2 testing reports with contact tracing data to compare completeness, reporting of contacts, and program timeliness.

**Results:** For August and October respectively, 4,600 (of 12,521) and 2,166 (of 16,269) individuals with COVID-19 were referred to the state program for case investigation. Investigators called 100% of referred individuals; 65% (August) and 76% (October) were interviewed. Of individuals interviewed, 33% reported contacts in August and 45% in October, with only mild variation by age, sex, race/ethnicity, and urbanicity. In August, 992 individuals with COVID-19 reported a total of 2,584 contacts (mean, 2.6), and in October, 739 individuals reported 2,218 contacts (mean, 3.0). Among contacts, 86% and 78% participated in interviews for August and October. The median time elapsed from specimen collection to contact interview was 4 days in August and 3 days in October, and from symptom onset to contact interview was 7 days in August and 6 days in October.

**Conclusions:** While contact tracing improved with time, the proportion of individuals disclosing contacts remained below 50% and differed minimally by demographic characteristics. The longest time interval occurred between symptom onset and test result notification. Improving elicitation of contacts and timeliness of contact tracing may further decrease SARS-CoV-2 transmission.

## Introduction

Case investigation and contact tracing are important tools to limit the spread of SARS-CoV-2, the virus causing coronavirus disease 2019 (COVID-19) (1). Efficient and timely case investigation and contact tracing, including isolating cases, identifying and quarantining contacts, and arranging testing for those contacts, can prevent ongoing transmission (2–5). Early isolation and/or quarantine can reduce SARS-CoV-2 transmission not only for symptomatic contacts, but also for contacts who develop asymptomatic infection when they might be unaware that they can transmit infection. Previous studies modeling contact tracing have demonstrated the importance of contact tracing coverage and quarantine and isolation rates and minimizing delays in testing symptomatic individuals (2–4).

With the rapid rise in COVID-19 infections, health departments swiftly expanded their contact tracing workforces, including recruiting staff who normally serve in other roles or staff without prior public health experience (6). Effective contact tracing requires staff trained in interviewing and rapport building, among other critical skills (7). Engagement and participation by individuals with COVID-19 and their contacts are also key factors. Health departments across the United States (US) have reported substantial challenges with individual engagement in the contact tracing process and significant variation in rates of contact disclosure (8–11). Underreporting of close contacts limits the effectiveness of contact tracing (5).

We previously found that lack of reporting of contacts was a significant barrier to contact tracing in central Washington, as nearly 70% of individuals with COVID-19 interviewed reported no close contacts (12). In this study, we broadened the geographic and demographic scope to include all individuals with COVID-19 investigated by the Washington State community contact tracing program during the study period. Our objective was to evaluate participation in contact tracing by individuals with COVID-19 and their contacts, how participation varied by demographic group, contact tracing timeliness, and the change in each of these measures over time.

## Methods

We retrospectively evaluated COVID-19 community case investigation and contact tracing surveillance data from Washington State for August 1–31, 2020 and October 1–31, 2020. Date ranges were chosen because case counts for Washington State were relatively stable during these periods (7-day rolling case averages were 728 and 989 on August 1 and October 31, 2020, respectively), prior to a sharp increase in cases during November 2020 (13).

In Washington State, some local health jurisdictions conduct all contact tracing locally, others primarily rely on the state program, and a third group uses the state contact tracing program for overflow capacity; jurisdictions can move between these categories depending on their capacity to conduct contact tracing at a given time. This study gathered data, including total COVID-19 case counts, only from jurisdictions that fell into the latter two groups. All cases referred to the state program came from local health jurisdictions directly, with each jurisdiction separately deciding which cases (and how many) to refer to the state program. Cases were defined as individuals with a positive SARS-CoV-2 nucleic acid amplification test (NAAT) or antigen test. Close contacts (hereafter “contacts”) were defined as individuals who had been within 6 feet of a person with COVID-19 for at least 15 minutes. Individuals diagnosed with COVID-19 were eligible for inclusion if they: (1) resided in Washington; (2) were referred to the state-run community case investigation and contact tracing program; (3) had a first positive test collected within the analysis period; and (4) had an available phone number. Individuals were excluded if contact tracing was done by the local health district, or if they resided in a long-term care facility or correctional/detention facility, since contact tracing at these facilities was handled by a separate team. Outreach to cases and contacts was conducted via telephone and text message. Before being classified as not having responded to phone calls, individuals diagnosed with COVID-19 received at least three phone calls, and contacts received at least two phone calls, with text message reminders following each. When reached, contact tracing staff conducted structured interviews with individuals with COVID-19 and their contacts, shared information about how to prevent the spread of COVID-19, and connected individuals to additional support services as needed (14).

SARS-CoV-2 NAAT and antigen testing data were collected from the Washington Disease Reporting System, the state's notifiable diseases surveillance system, and combined with case investigation and contact tracing surveillance data (stored in a separate database). We assessed demographic information, including urbanicity (defined using US Department of Agriculture Economic Research Service rural-urban commuting area codes, where values 1–3, 4–6, and 7 and above were considered metropolitan, micropolitan, and small town/rural, respectively) (15). We also evaluated completeness of initiating case investigation and contact tracing, participation in interviews by individuals with COVID-19 and contacts, the proportion of individuals naming contacts, and the median number of days and interquartile range (IQR) between each stage of the case investigation and contact tracing process. Measures for August and October were compared. We hypothesized that contact tracing performance measures would improve from August to October but sought to evaluate whether performance measures either improved or worsened. Chi-squared tests were performed for selected categorical variables and two-tailed *t*-tests for mean values comparing between months, with a statistical significance threshold of *p*-value < 0.05. Analyses were performed using R version 4.0.3 (R Foundation for Statistical Computing; Vienna, Austria).

## Results

The jurisdictions in Washington State that referred cases to the state contact tracing program recorded 12,521 cases of COVID-19 during August 1–31, 2020 and 16,269 during October 1–31, 2020 (Table 1). Of those, 4,600 (37%) and 2,166 (13%) cases in August and October, respectively, were referred to the state-run community case investigation and contact tracing program for investigation. Case and contact demographics are described in Table 1.

**Table 1:** Demographic information for individuals with COVID-19 and contacts—Washington State, August and October 2020.

**Measure**	**August**		**October**	
	***n*** **or Median**	**% or IQR^a^**	***n*** **or Median**	**% or IQR^a^**
**Cases**				
Total diagnoses of COVID-19^b^	12,521	–	16,269	–
Individuals with COVID-19 not referred to state contact tracing program^c^	7,921	63	14,103	87
Individuals with COVID-19 referred to state for case investigation	4,600	37	2,166	13
Age (median, years)	35	23–49	35	23–50
0–17 years	642	14	258	12
18–22 years	481	10	242	11
23–44 years	2,003	44	945	44
45–64 years	1,102	24	490	23
≥65 years	370	8	231	11
Unknown age	2	0	–	–
Female	2,145	47	989	46
Male	2,087	45	1,012	47
Unknown sex	368	8	165	8
Hispanic	1,954	42	715	33
Non-Hispanic white	902	20	694	32
Non-Hispanic black	129	3	80	4
Other or unknown race/ethnicity	1,615	35	677	31
Metropolitan^d^	3,911	85	1,690	78
Micropolitan^d^	407	9	362	17
Small town or rural^d^	263	6	104	5
Unknown urbanicity^d^	19	0	10	0
**Contacts**				
Total contacts elicited	2,584	–	2,218	–
Household contact	2,060	80	1,649	74
Non-household contact	504	20	567	26
Unknown household status	20	1	2	0
Age (median, years)	33	21–45	36	25–47
0–17 years	459	18	258	12
18–22 years	266	10	209	9
23–44 years	1,195	46	1,084	49
45–64 years	523	20	496	22
≥65 years	141	5	171	8
Female	1,326	51	1,097	49
Male	1,038	40	928	42
Unknown sex	220	9	193	9
Hispanic	1,512	59	984	44
Non-Hispanic white	756	29	827	37
Non-Hispanic black	76	3	78	4
Other or unknown race/ethnicity	240	9	329	15
Metropolitan^d^	1,943	75	1,431	65
Micropolitan^d^	235	9	407	18
Small town or rural^d^	148	6	101	5
Unknown urbanicity^d^	258	10	279	13

*IQR, Interquartile range.*

a *Percentages may not sum to 100% due to rounding.*

b *Represents the total diagnoses from all local health jurisdictions that referred cases to the Washington State community contact tracing program during August and October, respectively.*

c *Local health jurisdictions provided case investigation and contact tracing for these individuals (i.e., these individuals were not referred to the state for case investigation), and thus were not included in this analysis.*

d *Defined using US Department of Agriculture Economic Research Service rural-urban commuting area codes, where values 1 through 3, 4–6, and 7 and above were considered metropolitan, micropolitan, and small town or rural, respectively [see reference (14)]*.

Case investigators attempted to contact 100% of referred cases during both months (Table 2). In August, 65% of individuals with COVID-19 participated in case interviews, 12% refused interview, 19% did not answer contact attempts, and 3% had a non-working phone number. In October, 76% of individuals with COVID-19 participated in case interviews, 7% refused interview, 16% did not answer contact attempts, and 1% had a non-working phone number.

**Table 2:** Completeness of and participation in COVID-19 case investigation and contact tracing—Washington State, August and October 2020.

**Measure**	**August**		**October**		***p*** **-value^b^**
	***n***	**%^a^**	***n***	**%^a^**	
**Cases**					
Total diagnoses of COVID-19^c^	12,521	–	16,269	–	–
Individuals with COVID-19 referred to state for case investigation	4,600	37	2,166	13	<0.001
Individuals called by investigator	4,600	100	2,166	100	–
Individuals interviewed	3,000	65	1,639	76	<0.001
Individuals unable to be interviewed	1,600	35	527	24	
Refused	567	12	153	7	–
No response to phone calls	890	19	342	16	–
Non-working phone number	143	3	32	1	–
**Contacts**					
Total contacts elicited	2,584	–	2,218	–	–
Contacts listed per case (mean)	2.6	–	3.0	–	<0.001
Contacts interviewed	2,212	86	1,730	78	<0.001
Contacts unable to be interviewed	372	14	488	22	
Refused	77	3	78	4	–
No response to phone calls	295	11	410	18	–

a *Percentages may not sum to 100% due to rounding.*

b *Statistical testing is comparing categories or means between August and October.*

c *Represents total diagnoses from all local health jurisdictions that referred cases to the Washington State community contact tracing program during August and October, respectively*.

Table 3 displays the proportion of individuals with COVID-19 interviewed who reported contacts. Of individuals interviewed, 33% reported contacts in August and 45% in October. Stratified by urbanicity for August, the proportion reporting contacts was 33% for metropolitan residents, 35% for micropolitan residents, and 33% for rural residents in August. This increased to 43% for metropolitan residents, 53% for micropolitan residents, and 57% for rural residents in October. Stratified by race/ethnicity for August, 34% of Hispanic individuals, 37% of non-Hispanic White individuals, 25% of non-Hispanic Black individuals, and 26% of individuals of other or unknown race/ethnicity reported contacts. For October, this increased to 46% of Hispanic individuals, 48% of non-Hispanic White individuals, and 34% of non-Hispanic Black individuals, and 40% of individuals of other or unknown race/ethnicity. The proportion of individuals reporting contacts improved across all age and birth sex categories from August to October.

**Table 3:** Reporting of contacts by individuals with COVID-19—Washington State, August and October 2020.

**Measure**	**August**		**October**		
	***n***	**%**	***n***	**%**	***p*** **-value^a^**
Individuals interviewed^b^	3,000	65	1,639	76	<0.001
Individuals with COVID-19 reporting contacts^c^	992	33	739	45	<0.001
0–17 years	149	34	83	42	0.05
18–22 years	95	31	72	40	0.04
23–44 years	435	33	339	47	<0.001
45–64 years	239	33	172	45	<0.001
≥65 years	74	34	73	47	0.01
Female	481	34	347	46	<0.001
Male	424	32	331	44	<0.001
Hispanic	563	34	300	46	<0.001
Non-Hispanic white	301	37	301	48	<0.001
Non-Hispanic black	30	25	26	34	0.18
Other or unknown race/ethnicity	98	26	112	40	<0.001
Metropolitan^d^	837	33	550	43	<0.001
Micropolitan^d^	88	35	144	53	<0.001
Small town or rural^d^	63	33	42	57	<0.001

a *Statistical testing is comparing categories or means between August and October.*

b *Denominator is the total number of individuals with COVID-19 referred for case investigation.*

c *Denominators for reporting of contacts and corresponding rows below it are the total number of individuals interviewed, by respective demographic group where applicable.*

d *Defined using US Department of Agriculture Economic Research Service rural-urban commuting area codes, where values 1–3, 4–6, and 7 and above were considered metropolitan, micropolitan, and small town or rural, respectively [see reference (14)]*.

Overall, 992 individuals with COVID-19 reported 2,584 contacts (mean 2.6 contacts per person; median 2 contacts, IQR 1–3) in August, and 739 individuals with COVID-19 reported 2,218 contacts (mean 3.0 contacts per person; median 2 contacts, IQR 1–4) in October (Table 2). Eighty percent of reported contacts were household contacts in August, decreasing to 74% in October (Table 1). Of all contacts reported, 86 and 78% participated in contact tracing interviews for August and October, respectively (Table 2).

Regarding timeliness of contact tracing, the median interval from case symptom onset to specimen collection was 3 days (IQR 1–5) for August and 2 days (IQR 1–5) for October. Specimen collection to when the positive result was received by the state department of health (hereafter referred to as “state notification of a positive result”) was 2 days (IQR 2–3) for August and 2 days (IQR 1–2) for October; from state notification of a positive result to case interview, 1 day (IQR 1–2) for both months (Table 4). A median of 0 days (IQR 0–0 for August, 0–1 for October) elapsed between case interview and contact interview (i.e., most contacts were interviewed the same day they were reported as a contact) for both months. Overall, the median duration from specimen collection to contact interview was 4 days (IQR 3–5) in August and 3 days (IQR 2–4) in October and from case symptom onset to contact interview was 7 days (IQR 5–10) in August and 6 days (IQR 4–8) in October.

**Table 4:** Case investigation and contact tracing timeliness measures—Washington State, August and October 2020.

**Measure**	**August**		**October**	
	**Median**	**IQR**	**Median**	**IQR**
**Case timeline**				
Symptom onset to specimen collection, days	3	1–5	2	1–5
Specimen collection to state notification, days	2	2–3	2	1–2
State notification to interview, days	1	1–2	1	1–2
Specimen collection to interview date, days	4	3–5	3	2–4
Symptom onset to interview date, days	7	5–10	6	4–8
**Contact timeline**				
Case specimen collection to contact interview, days	4	3–5	3	2–4
Case interview to contact interview, days	0	0–0	0	0–1
Case symptom onset to contact interview date, days	7	5–10	6	4–8

*IQR, interquartile range*.

## Discussion

From August to October 2020, the proportion of individuals with COVID-19 who were interviewed increased from 65 to 76%, and among individuals interviewed, those reporting contacts increased from 33 to 45%. More than 75% of contacts participated in an interview during both analysis periods, likely because most contacts were household members. Staff reached cases and contacts efficiently, reaching most individuals with COVID-19 within 1 day of state notification of a positive result and reaching contacts on the same day that an individual with COVID-19 disclosed their information. The longest time intervals occurred between case symptom onset to specimen collection and specimen collection to state notification of a positive result.

Effective contact tracing is predicated on completeness (tracing as many contacts as possible) and timeliness (reaching contacts as quickly as possible following exposure), among other factors. One recent COVID-19 modeling study suggested that contact tracing occurring within 4.5 days of exposure could achieve at least a 60% reduction in transmission (16). It also predicted minimal benefits in transmission reduction when contacts are reached more than 6.5 days after first exposure. While date of first exposure to an individual with COVID-19 was not available in our study, the delay between case symptom onset to contact interview (i.e., when the contact is first notified of their exposure and need to quarantine) suggests that at least some contacts may not be reached soon enough for contact tracing to effectively reduce onward transmission. For contact tracing programs facing delays from symptom onset to SARS-CoV-2 testing (the longest step of this analysis), continuing to emphasize the importance of seeking testing as soon as potential COVID-19 symptoms develop, especially for unvaccinated individuals, may be beneficial.

There are multiple potential reasons for the increased participation in case interviews and elicitation of contacts through contact tracing observed over time. First, while the overall COVID-19 case count for Washington remained relatively stable from August through October, the absolute number of cases referred to the state program decreased by over 50%, likely due to multiple factors. For example, local health jurisdictions expanded contact tracing capacity as the pandemic progressed, and universities initiated contact tracing programs as the school year began in the early fall. Both may have contributed to a decreased reliance on the state program to handle similar caseloads, potentially facilitating more time for state case investigators to spend conducting case interviews. Second, this may reflect increasing contact tracer proficiency. In September, the Washington State Department of Health began the transition to a permanent contractor workforce for case investigation and contact tracing. This transition to a more stable workforce may have facilitated trusting and supportive connections with individuals with COVID-19.

Though participation in the contact tracing process improved, the high proportion of individuals that did not report any contacts remains a major barrier to effective contact tracing. Across various US jurisdictions, published data on the proportion of cases not naming contacts ranged from 35 to 83% (8–12). Publicly available COVID-19 dashboards from Maryland and New Jersey indicate similar variability, 18 and 60%, respectively (17, 18). One public opinion survey found that 41% of US adults would not be likely to participate in contact tracing and nearly 30% would not feel comfortable sharing information about contacts (19). Participation in contact tracing and reporting of contacts are voluntary in the US (20, 21). Prior studies of other infectious diseases indicate wide variability among the proportion of cases reporting contacts (HIV, 31–61%; syphilis 42%; tuberculosis, 76–91%) (22–27).

While the true number of contacts per individual with COVID-19 in Washington is unknown, only 27% of Washington state households consist of one person living alone, and the average household size is 2.5 (28). Thus, the low proportion of individuals reporting contacts likely reflects underreporting. Among potential explanations for underreporting of contacts, contact tracing exclusively via telephone outreach might limit rapport building and trust of individuals with COVID-19. At a societal level, trust in all levels of government has decreased as the pandemic has progressed, and contact tracers may be seen as representing the government (29). Concerns about data privacy and misuse and naming family or friends as contacts may also discourage reporting (30). Although underreporting is most likely, community mitigation efforts may have decreased the number of individuals with contacts and the mean number of contacts per individual with COVID-19. For example, Washington State implemented recommendations for when K-12 schools should consider remote learning and a county-by-county phased reopening plan that included limits on workplace occupancy, size of gatherings, and recreation (31, 32).

In our study, elicitation of contacts improved over time across all demographic groups. There was mild variation by race/ethnicity across time, with non-Hispanic Black individuals reporting contacts at the lowest levels for August and October, and by urbanicity, as individuals residing in metropolitan areas reported contacts at lower rates during October. Small town and rural residents accounted for the largest absolute increase in contact reporting rates through October, with a 24 percentage-point increase. Reporting of contacts did not differ substantially by age and sex. Strategies to improve contact tracing may require tailoring outreach and implementation to the unique concerns of each community.

Approaches to overcome barriers to contact tracing and improving trust and engagement include (1) culturally appropriate and locally tailored messaging that raises awareness and encourages participation, (2) partnering with trusted community and health care organizations to spread key messages, (3) incentivizing participation, and (4) providing ongoing training to contact tracers (1, 33). Additionally, strengthening social and financial protections for individuals who contract COVID-19 or who must quarantine as a contact could reduce financial hardship and promote greater participation in contact tracing (1, 34, 35).

This study is subject to a few limitations. First, given the scope of the programmatic data collected, we cannot assess why individuals did not participate in interviews or report contacts, limiting insight into potential barriers to effective contact tracing. Additionally, we cannot evaluate whether individuals isolated or quarantined, limiting greater insight into the impact of case investigation and contact tracing, or whether contacts subsequently tested positive for SARS-CoV-2. Third, date of symptom onset was missing for 38% of cases referred to the state, primarily among individuals who could not be reached for case investigation, and thus timeliness measures from symptom onset to other endpoints do not include those individuals for whom symptom onset date was missing. Race/ethnicity data was also missing for 28% and 23% of individuals in August and October, respectively, which may limit comparisons across racial/ethnic groups. Finally, these data only represent cases investigated by the Washington State Department of Health centralized program and do not include cases investigated by local jurisdictions, which may limit the generalizability of our conclusions.

This study demonstrates that while contact tracing improved during a period of stable COVID-19 case counts, the proportion of individuals disclosing contacts remained below 50%, with minimal differences by demographic characteristics. The contact tracing program efficiently reached individuals with COVID-19 and their contacts for interview, but the longest time interval occurred between symptom onset and state notification of a positive result. Improving elicitation of contacts and the timeliness of contact tracing may further decrease SARS-CoV-2 transmission.

## Data Availability Statement

The raw data supporting the conclusions of this article will be made available by the authors, without undue reservation.

## Ethics Statement

Ethical review and approval was not required for the study on human participants in accordance with the local legislation and institutional requirements. Written informed consent from the participants' legal guardian/next of kin was not required to participate in this study in accordance with the national legislation and the institutional requirements.

## Author Contributions

RB and JM conceived and designed the study. LM conducted data analysis. RB, LM, JM, PM, ML, LMD, LBD, RL, and JO contributed to interpretation of the data. RB drafted the initial manuscript. RB, LM, JM, PM, ML, LMD, LBD, RL, and JO revised the manuscript critically for important intellectual content. RB, PM, and JO integrated all feedback from required clearance at CDC prior to publication. All authors contributed to the article and approved the submitted version.

## Author Disclaimer

The findings and conclusions in this report are those of the authors and do not necessarily represent the official position of the Centers for Disease Control and Prevention.

## Conflict of Interest

The authors declare that the research was conducted in the absence of any commercial or financial relationships that could be construed as a potential conflict of interest.

## Publisher's Note

All claims expressed in this article are solely those of the authors and do not necessarily represent those of their affiliated organizations, or those of the publisher, the editors and the reviewers. Any product that may be evaluated in this article, or claim that may be made by its manufacturer, is not guaranteed or endorsed by the publisher.
